# An In Situ Gelling System for the Local Treatment of Inflammatory Bowel Disease (IBD). The Loading of Maqui (*Aristotelia chilensis*) Berry Extract as an Antioxidant and Anti-Inflammatory Agent

**DOI:** 10.3390/pharmaceutics11110611

**Published:** 2019-11-14

**Authors:** Marika Tenci, Silvia Rossi, Valentina Giannino, Barbara Vigani, Giuseppina Sandri, Maria Cristina Bonferoni, Maria Daglia, Luigi Maria Longo, Cristina Macelloni, Franca Ferrari

**Affiliations:** 1 Department of Drug Sciences, University of Pavia, V.le Taramelli, 12, 27100 Pavia, Italy; marika.tenci.88@hotmail.it (M.T.); barbara.vigani@unipv.it (B.V.); giuseppina.sandri@unipv.it (G.S.); cbonferoni@unipv.it (M.C.B.); franca.ferrari@unipv.it (F.F.); 2 Cosmo SpA; Via C. Colombo 1, 20020 Lainate (MI), Italy; VGiannino@cosmopharma.com (V.G.); CMacelloni@cosmopharma.com (C.M.); 3 Department of Pharmacy, University of Naples Federico II, Via Domenico Montesano, 49, 80131 Napoli, Italy; maria.daglia@unina.it; 4 International Research Center for Food Nutrition and Safety, Jiangsu University, Zhenjiang 212013, China; 5 Cosmo Pharmaceuticals NV, Riverside II, Sir Rogerson’s Quay, Dublin 2, Ireland; llongo@cosmopharma.com

**Keywords:** in situ gelling systems, thermo-responsive polymers, ion-sensitive polymers, methylcellulose, gellan, mucoadhesion, DoE approach, maqui berry extract, antioxidant properties, inflammatory bowel disease

## Abstract

The aim of the present work was the development of an innovative in situ gelling system, to be applied on the mucosa of the distal colon via rectal route. The system consisted of three polymers having different functions: gellan (GG), able to jellify in presence of ions; methylcellulose (MC), a thermosensitive polymer with a gelation temperature close to 50 °C; and hydroxypropylcellulose (HPC), a mucoadhesive polymer. The three polymers were able to act synergistically, increasing the permanence of the vehicle on the mucosa and forming a protective gel layer. A DoE approach, “simplex centroid mixture design,” was used to identify the optimal quantitative composition of the vehicle. The response variables considered were: vehicle viscosity at room temperature; increase in vehicle viscosity on increasing temperature (from room to physiological value) and upon dilution with simulated colonic fluid (SCF); and viscoelastic behavior, thixotropic area, and mucoadhesion properties of the gel formed at 37 °C upon dilution in SCF. The optimized vehicle was loaded with maqui berry extract (MBE), known for its antioxidant and anti-inflammatory properties. MBE loading (0.5% *w*/*w*) into the vehicle improved rheological and mucoadhesive properties of the formulation. Both MBE and the optimized vehicle were not cytotoxic towards human fibroblasts and Caco-2 cells. Moreover, the optimized vehicle did not affect MBE antioxidant properties.

## 1. Introduction

Ulcerative colitis (UC) and Crohn’s disease (CD) are two subtypes of inflammatory bowel disease (IBD); that is, a relapsing-remitting inflammation of the gastrointestinal tract. IBD is rapidly growing worldwide, especially in young people within industrialized countries [1]. In particular, UC refers to an inflammatory disease regarding the mucosa and the submucosa of the large intestine. Even though the cause of UC is still unclear, it is generally recognized that it arises from a complex interaction between genetic, immune-regulatory, and environmental factors. Among the environmental factors, oral contraceptives, breast-feeding, infections, microorganisms, smoking, appendectomies, sanitation, and stress seem to play important roles [1,2,3,4]. The clinical picture of UC encompasses several intestinal manifestations, like abdominal pain, bloody stool, and diarrhea. Extra-intestinal manifestations are also frequent. They may involve the skin and eyes, but also the liver, lungs, and pancreas [1,4].

The treatment of UC depends on the severity of the disease and on patient-related factors, such as pre-existing illnesses, adherence, and compliance towards the therapy [5].

Actually, the mainstay of UC treatment still consists of the so-called conventional therapies, including the use of aminosalicylates (i.e., mesalazine and olsalazine), corticosteroids (i.e., prednisolone), and immune-modulatory agents (i.e., azathioprine) [1,5,6].

Surgery is considered a therapeutic strategy for UC only when patients fail to adequately respond to the pharmacological therapies, such as in case of refractory and fulminant diseases, or in the case of complications [7].

Many of the above-mentioned drugs result in from mild to severe adverse reactions, including mortality [1]. The development of innovative therapeutic systems, characterized by a suitable balance between therapeutic efficacy and side effects, represents a stimulating challenge for pharmaceutical technology scientists [8].

In the last decade, in situ gelling systems intended for local administration were proposed as an alternative to conventional dosage forms [9,10,11,12,13,14,15]. Such systems, based on stimuli-responsive polymers, also called “smart” materials, do not require organic solvents or copolymerization agents [16]. Before administration, they are liquid, aqueous solutions that are able to jellify in physiological conditions. In fact, in situ sol-gel transition occurs as a response of the contact of polymers with biological fluids, rich in ions, or of a change in pH or temperature [17,18,19].

Among thermo-responsive polymers, some cellulose derivatives, like methylcellulose (MC) and hydroxylpropylmethylcellulose (HPMC), have been investigated [20,21,22]. In particular, a gelling temperature (*T*_g_) higher than 50 °C has been reported in the literature for MC. In the past, many attempts have been made to reduce MC *T*_g_ to the physiological range. From that perspective, some authors demonstrated the influence of various water-soluble polymers, such as polyvinylpyrrolidone, on MC *T*_g_ [23]. The effects of pH, salts, and bioceramics on MC *T*_g_ have been also investigated [23,24,25,26].

Other interesting “smart” materials are ion-sensitive polymers, including gellan gum (GG) and k-carrageenan (K CARR), which may undergo sol-gel transitions in the presence of specific ions [13,14,27,28,29].

There is strong evidence in the literature of the beneficial effects on health of a diet rich in polyphenols. Recently, some authors demonstrated that polyphenols, like anthocyanins, exhibit protective and therapeutic effects on IBD. In particular, they do not only show antioxidant activities, but also exert a down-regulation of inflammatory mediators, such as cytochines, induce a suppression of inflammatory pathways, and preserve tight junctions [30,31,32,33,34,35]. Fruits of *Aristotelia chilensis*, known as maqui and grown in Chile and Argentina, represent a rich source of polyphenolic compounds [36]. The increasing interest in maqui fruit, commonly known as the Chilean wine-berry, has been attributed to the phytochemical profile of berry extracts (MBE) and their antioxidant and anti-inflammatory properties, which are strongly related to anthocyanin content [37,38,39]. Many studies dealing with in vitro and in vivo biological activities of MBE have demonstrated its efficacy as an antioxidant, antimicrobial, and anti-inflammatory agent [39,40,41,42,43].

Given these premises, the aim of this work was the development of an innovative, in situ gelling system, to be applied on the mucosa of the distal colon via rectal route by means of a rectal tube [44].

Recently, some authors have underlined the relevance of combinations of mucoadhesive and in situ gelling polymers to keep the delivery systems at the target site for as long as possible [45].

Keeping this in mind, three hydrophilic polymers were accurately selected as functional materials: two of them were meant to enable in situ gelation according to different mechanisms (presence of ions and temperature increase), while the third one was supposed to possess mucoadhesive properties. The polymers should act synergistically to increase the contact time of the formulation with the mucosa, forming a protective gel layer on it.

The research was articulated in two phases: the first one was devoted to the choice of the three polymers, which were the components of the vehicle, and of their concentrations. GG and K CARR were investigated as alternative ion-sensitive polymers; methylcellulose (MC) was studied as a thermosensitive gelling agent; and hydroxypropylcellulose (HPC) was used as mucoadhesive polymer. A DoE approach, “simplex centroid mixture design,” was employed to identify the optimal quantitative composition of the vehicle. The response variables considered were: (i) vehicle viscosity at room temperature; (ii) increase in vehicle viscosity resulting from temperature increase (from room to physiological value) and from the dilution with simulated colonic fluid (SCF); (iii) viscoelastic behavior, at 37 °C, of the gel upon dilution in SCF; (iv) diluted gel thixotropic area at 37 °C; and (v) diluted gel mucoadhesive properties at 37 °C.

In the second phase of the research, the vehicle of optimal composition identified in the first phase, was loaded with MBE obtained from the maqui fruit. Antioxidant activity of the loaded vehicle was tested in vitro on human colorectal adenocarcinoma (Caco-2) cell lines.

## 2. Materials and Methods

### 2.1. Materials

The following materials were used: acetonitrile, HPLC-grade (Chromasolv, Sigma Aldrich, Milan, Italy); antibiotic anti-mycotic solution (100×), stabilized with 10,000 penicillin units, 10 mg streptomycin, and 25 μg amphotericin B per mL (Sigma Aldrich, Milan, Italy); calcium chloride (Sigma Aldrich, Milan, Italy); cyanidin 3 glucoside (PhytoLab GmbH and Co. KG, Vestenbergsgreuth, Germany); Dulbecco’s Modified Eagle Medium (DMEM; Lonza, Walkersville, MD, USA); Dulbecco’s phosphate buffer solution (Sigma Aldrich, Milan, Italy); formic acid 1M (Bioultra, Sigma Aldrich, Milan, Italy); gellan gum with low acetylation degree (GG; Kelcogel^®^; Giusto Faravelli, Milan, Italy); Hank’s balance salt solution (HBSS; Sigma Aldrich, Milan, Italy); HCl 1M (Carlo Erba, Milan, Italy); inactivated fetal calf bovine serum (Euroclone, Pero, Italy); k-carrageenan (K CARR; FlukaBioChemika, Buchs, Switzerland); KCl (Carlo Erba, Milan, Italy); KH_2_PO_4_ (Carlo Erba, Milan, Italy); maqui powder from dehydrated berries (CiboCrudo, Milan, Italy); methanol HPLC-grade (Chromasolv, Sigma Aldrich, Milan, Italy); Methocel A4M (Colorcon Ltd., Dartford, Kent, UK); MTT (3-(4,5-dimethylthiazol-2-yl)-2,5-diphenyltetrazolium bromide) (Sigma Aldrich, Milan, Italy); gastric porcine mucin (Sigma Aldrich, Milan; Italy); NaCl (Panreac, Milan, Italy); Na_2_HPO_4_ (Carlo Erba, Milan, Italy); trypan blue solution (Biological Industries, Beit-Haemek, Israel) and trypsin–EDTA solution (Sigma Aldrich, Milan, Italy).

### 2.2. Choice of the Gelling Agent

The ability of either GG or K CARR to jellify thanks to the ions of the colonic fluid, was investigated. GG and K CARR aqueous solutions (1.4% *w*/*w*) were diluted in simulated colonic fluid (SCF) according to a 5:2 weight ratio. Schiller et al. [46] investigated the availability of fluid along the human intestinal lumen by magnetic resonance imaging and found that the mean fluid volumes in the colon were 13 ± 12 mL in the fasting state and 18 ± 26 mL after a meal. On the basis of such results, the 5:2 *w*/*w* ratio was chosen to plan a range of formulation volumes to be administered from 30 to 50 mL. SCF was prepared according to Marques et al. [47]: KCl 0.2 g/L, NaCl 8 g/L, KH_2_PO_2_ 0.24 g/L, and NaHPO_4_ g/L. pH was adjusted to 6.3 by adding HCl 1 M.

Upon dilution, the solutions were subjected to viscosity measurements by using a rotational rheometer (Rheostress 600, Thermo Fisher Scientific, Waltham, MA, USA), equipped with a cone plate (C60/1: Ø 60 mm; angle = 1°) combination as the measuring system.

Viscosity was measured at increasing shear rates (ranging from 10 up to 300 s^−1^) at a constant temperature (37 °C). In order to evaluate the ion-sensitive gelling properties of the two polymers, viscosity profiles of the polymer solutions diluted in SCF were compared with those obtained for the same solutions diluted in distilled water. The results were expressed as normalized Δ viscosity (Δη%), calculated, at each shear rate, as follows:

Δη% = ((η_SCF_ − η_DW_)/η_DW_) × 100, (1)

where η_SCF_ is the viscosity (Pa·s) at 37 °C of the polymer solution diluted 5:2 *w*/*w* in SCF and η_DW_ is the viscosity at 37 °C (Pa·s) of the polymer solution diluted 5:2 *w*/*w* in distilled water.

### 2.3. Influence of GG on MC Rheological Properties

To evaluate the effect of GG on MC rheological properties, a 1:1 *w*/*w* mixture of MC (1% *w*/*w*) and GG (1% *w*/*w*) aqueous solutions was subjected to oscillation measurements in comparison with an MC solution prepared at the same concentration present in the mixture. Before the analysis, both a GG/MC mixture and an MC solution were diluted 5:2 *w*/*w* in SCF. Dynamic viscoelastic measurements (oscillation test) were performed by the rotational rheometer mentioned in Section 2.2 equipped with a C20/1 cone plate measuring system. A constant shear stress, chosen in the linear viscoelastic region previously determined by means of a stress sweep test, was applied at a fixed frequency (1 Hz) and at increasing temperatures ranging from 20 to 42 °C. G’ (storage modulus), which is an index of sample elasticity, was recorded as a function of temperature.

### 2.4. DoE Approach: Simplex Centroid Mixture Design

The DoE approach is widely applied to optimize the composition of mixtures, i.e., excipients, in pharmaceutical products manufacturing [48,49]. For a three-component mixture, the experimental space is a triangular plane and three different experimental designs with three, six, seven, or ten points can be selected [48,49,50,51]. The goal of a mixture design is to determine an optimum blend of components (factors) inside the experimental region, characterized by the desired values of each response variable considered [52].

**Figure 1:**
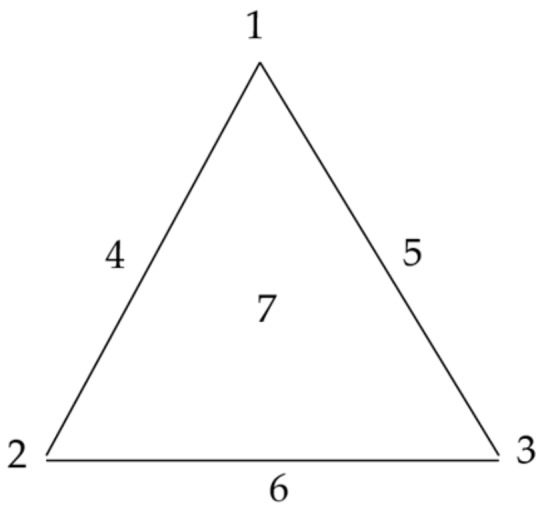
Factor space and experimental points of a simplex centroid mixture design.

In this work, a “simplex centroid mixture design” with 7 points was applied. When three factors (*k*) are considered, 7 (*n* = 2*^k^* − 1) experiments have to be carried out. Such a mixture design includes: three pure components, corresponding to the triangle corners; three binary combinations, graphically represented as points in the middle of the triangle sides; and one ternary combination, that corresponds to the center of the triangle [53]. Three aqueous stock solutions: GG 0.8% *w*/*w*, MC 1% *w*/*w*, and HPC 1% *w*/*w* were prepared. These solutions were used as such or mixed in predetermined weight ratios to obtain the seven points of the simplex centroid mixture design, corresponding to the characteristic points of a Sheffè triangle (Figure 1 and Table 1).

#### 2.4.1. Vehicle Characterization

The vehicles corresponding to the different experimental points of the “simplex centroid mixture design” were characterized for rheological (viscosity, viscoelasticity, and thixotropic area) and mucoadhesive properties, as described hereafter.

##### Rheological Properties

Rheological properties were evaluated by means of the rotational rheometer, mentioned in Section 2.2, equipped with cone plate measuring systems (C20/1 or C60/1).

Vehicles were subjected to viscosity measurements at 25 °C by applying 10 s^−1^ shear rate. Moreover, each vehicle was diluted 5:2 *w*/*w* in distilled water or SCF and subjected to viscosity measurements. Viscosity measurements were performed at 25 °C for the vehicles diluted in water and at 37 °C for the vehicles diluted in SCF. The results were expressed as: (i) the viscosity of undiluted vehicles at 25 °C with a 10 s^−1^ shear rate and (ii) the normalized interaction parameter (ΔIp). ΔIp was calculated as follows:

ΔIp = (η_SCF, 37 °C_ − η_DW, 25 °C_)/η_DW, 25 °C_, (2)

where η_SCF, 37 °C_ = viscosity at 37 °C of the vehicle diluted in SCF and η_DW, 25 °C_ = viscosity at 25 °C of the vehicle diluted in distilled water.

Vehicles diluted 5:2 *w*/*w* in SCF were also subjected to thixotropy measurements: increasing (10–300 s^−1^; up-flow curve) and decreasing (300 s^−1^–10 s^−1^; down-flow curve) shear rates were applied at a constant temperature (37 °C). The results were expressed as thixotropic area (TA _37 °C_), also called hysteresis area, which was calculated by the following equation:

TA _37 °C_ = *A*_up_ − *A*_down_, (3)

where *A*_up_ and *A*_down_ are the areas under the up-flow and down-flow curves, respectively.

Upon 5:2 *w*/*w* dilution in water or SCF, vehicles were also subjected to oscillation measurements at 37 °C and at 1 Hz frequency, in order to measure the loss modulus (G″), which is an index of viscous behavior, and the storage one (G′). The results were expressed as loss tangent, calculated as the ratio between G” and G’, at 37 °C and 1 Hz.

##### Mucoadhesive Properties

The mucoadhesive properties of the vehicles were assessed at 37 °C by means of a TA-XT plus Texture Analyzer (Stable Mycro Systems, Godalming, UK), equipped with 1 kg load cell and with a cylindrical movable probe (P/10C). Gastric porcine mucin was used as biological substrate. An 8% (*w*/*w*) mucin dispersion in SCF was prepared. Vehicles were diluted 5:2 *w*/*w* in SCF before testing. Each sample (40 mg) was layered on a filter paper disc (2 cm^2^) and fixed on the movable probe. A total of 40 µL of gastric mucin dispersion was fixed, facing to the formulation, on the sample holder. The probe was lowered to put the mucin dispersion in contact with the diluted vehicle. A preload of 2500 mN was applied for 180 s [54]. Then, the probe was raised at a constant speed (2.5 mm/s) up to the complete separation of the two surfaces. Blank measurements were also carried out using 40 µL of SCF instead of mucin dispersion.

The normalized work of adhesion (ΔAUC/AUC _37 °C_) parameter was calculated according to the following equation:

ΔAUC/AUC _37 °C_ = (AUC_mucin_ − AUC_blank_)/AUC_blank_, (4)

where AUC_mucin_ is the adhesion work obtained in presence of gastric mucin dispersion, whereas AUC_blank_ is the adhesion work obtained from blank measurements.

### 2.5. Optimization Procedure

For each vehicle, the following response variables were considered: (i) viscosity of the undiluted vehicle at 25 °C (η _25 °C_); (ii) normalized interaction parameter ΔIp; (iii) loss tangent at 37 °C of the vehicle diluted in SCF (tgδ _37 °C_); thixotropic area at 37 °C of the vehicle diluted in SCF (TA _37 °C_); and normalized work of adhesion at 37 °C of the vehicle diluted in SCF (ΔAUC/AUC _37 °C_).

Each response variable could be related to the mixture composition according to a suitable mathematical model that adequately describes such a relation. Model selection was effected by means of a statistical software package (Statgraphics 5.0, Statistical Graphics, Rockville, MD, USA).

Experimental data were treated according to a multiple regression analysis, testing a series of models including linear, quadratic, and special cubic. The best fit model was chosen on the basis of statistical parameters such as F-ratio for significance of regression and adjusted correlation coefficient for the goodness of fit of the model [55,56].

The vehicle of optimized composition, chosen on the basis of the results of the experimental design, was prepared and subjected to the same characterization previously effected on the seven vehicles of the simplex centroid mixture design.

### 2.6. Maqui Berry Extract (MBE) Preparation

Maqui powder, from dehydrated berries, was submitted to solid–liquid extraction to obtain an extract rich in anthocyanins. In brief, one portion (1 g) was extracted in an ice bath, protected from light, under constant stirring and a nitrogen atmosphere, with 20 mL of an aqueous methanol mixture (H_2_O:MeOH/10:90 *v*/*v*, containing 0.1% formic acid 1 M). After 30 min, the extract was centrifuged at 8000 rpm for 10 min at room temperature. The extraction procedure was repeated three times. The three extracts were collected, filtered under vacuum on a 0.45 μm cellulose membrane filter (Sartorius Stedim Biotech GmbH, Gottingen, Germany), and freeze-dried at 8 × 10^−1^ mbar and −50 °C (Modulyo^®^ Edwards Freeze-Drier, Kingston, NY, USA).

### 2.7. Preparation and Characterization of MBE-Loaded Optimized Vehicle (MBE-VH)

MBE was solubilized under stirring in a GG/MC/HPC solution. The final polymer concentrations in MBE-VH were equal to those of the optimized vehicle. MBE concentration in the vehicle was 0.5% *w*/*w*.

MBE-VH was characterized for rheological and mucoadhesive properties as described for unloaded vehicles of the simplex centroid mixture design.

#### 2.7.1. Anthocyanin Assay in MBE and MBE-Loaded Optimized Vehicle (MBE-VH)

RP-HPLC-PDA analysis was performed using a ThermoFinnigan Surveyor Plus HPLC, equipped with a quaternary pump and a Surveyor UV−Vis diode array detector (Thermo Fisher Scientific, Waltham, MA, USA). A Synergi Fusion RP-18 column (150 × 4.6 mm, 5 μm), equipped with a Hypersil Gold C18 pre-column (10 × 2.1 mm, 5 μm), both from Phenomenex (Torrance, CA, USA), was used. The mobile phase consisted of water acidified with 2% *w/v* formic acid (eluent A) and acetonitrile acidified with 0.8% *w/v* formic acid (eluent B) and was eluted according to a suitable gradient program—0–5 min 2% B, from 2% to 25% B in 65 min, and from 25% to 100% B in 10 min, followed by a 5 min isocratic run of 100% B. Total run time was 92 min, including column reconditioning. The flow rate was 0.5 mL/min, and the auto-sampler and column temperatures were maintained at 4 and 25 °C, respectively. The injection volume was set to 5 µL. The chromatograms were registered at 254, 280, and 520 nm; spectral data were collected within the range of 200−800 nm for all peaks.

The analytical method was validated according to ICH (International Council for Harmonisation) procedures to demonstrate its suitability to quantify anthocyanins in MBE and in the formulation developed as cyanidin-3-glucoside (C3G) equivalents (ICH Harmonised Tripartite Guideline; Validation of analytical procedures: text and methodology (http://www.ich.org/fileadmin/Public_Web_Site/ICH_Products/Guidelines/Quality/Q2_R1/Step4/Q2_R1__Guideline.pdf (2012 ICH)). The results are expressed as the relative standard deviation percentage of the measurements (RSD%). Method sensibility was determined as limit of quantification (LOQ) and limit of detection (LOD).

The validated RP-HPLC-PDA method was applied both to quantify anthocyanin content in MBE and to estimate the MBE incorporation in the vehicle, expressed as percentage of C3G loaded. The formulation was properly diluted with Milli-Q grade water to reduce its viscosity before the analysis. The results were expressed as percentages of loaded C3G as the means of 4 different samples collected in different portions of the loaded formulation, each one analyzed in triplicate.

#### 2.7.2. In Vitro Evaluation of Biocompatibility and Antioxidant Properties of MBE-VH

Normal human dermal fibroblasts (NHDF; Promocell GmbH, Heidelberg, Germany) from 6th to 16th passage and human colorectal adenocarcinoma cell lines (Caco-2), obtained from the American Type Culture Collection (ATCC, Manassas, VA, USA), were used.

Fibroblasts and Caco-2 cells were cultured in polystyrene flasks (Greiner bio-one, PBI International, Milan, Italy) with 13–15 mL of Dulbecco’s modified eagle medium (DMEM) containing 4.5 g/L glucose and l-glutamine and supplemented with 1% (*v*/*v*) antibiotic anti-mycotic solution (100×), stabilized with 10,000 units penicillin, 10 mg streptomycin, and 25 μg amphotericin B per mL; 10% (*v*/*v*) inactivated fetal calf serum; and 1% (*v*/*v*) non-essential amino acids.

Cells were maintained in incubator (Shel lab^®^ Sheldon^®^ Manufacturing Inc., Cornelius, OR, USA) at 37 °C with a 95% air and 5% CO_2_ atmosphere. All the operations required for cell culture were carried out in a vertical laminar air flow hood (Ergosafe Space 2, PBI International, Milan, Italy). When cells had reached 80–90% confluence in the flask, a trypsinization was performed as described by Tenci et al. [57]. The number of viable cells after trypsinization was determined in a counting chamber (Hycor Biomedical, Garden Grove, CA, USA), using 0.5% (*w*/*v*) trypan blue solution.

##### MBE and MBE-VH Biocompatibility

To assess the biocompatibility of MBE and of unloaded (VH) and MBE-loaded optimized vehicle (MBE-VH) towards human fibroblasts and Caco-2 cells, cell viability tests were performed. Such tests were used to estimate the number of viable cells after contact for a prefixed time with the samples considered. Sterile distilled water was used for sample preparation and all the operations were performed in a vertical laminar air flow hood (Ergosafe Space 2, PBI International, Milan, Italy). In total, 3.5 × 10^4^ cell/well (0.35 cm^2^ area) were seeded in a 96-well plate (Greiner bio-one, VWR International, rectal tube Milan, Italy) with culture medium (CM) and let to grow to reach confluence. When the medium was removed, cells were put in contact with 200 μL of each sample.

Dependently on each cell line, different experimental conditions were considered: dilution medium (complete medium (CM) or pH 7.4 HBSS) and contact time (24 or 2 h) of cells with samples.

As for fibroblasts, samples were first diluted in CM and then put in contact with cells for 24 h. In the case of Caco-2, samples were diluted in pH 7.4 HBSS and 2 h was the contact time.

An MBE (0.5% *w*/*w*) solution in CM or HBSS was prepared and then diluted with CM or HBSS according to different *v/v* ratios (1:2, 1:5, 1:10, 1:20, and 1:40). Biocompatibility of the undiluted 0.5% *w*/*w* MBE was also investigated. VH and MBE-VH were diluted with CM or HBSS according to different *v/v* ratios (1:2, 1:5, 1:10, 1:20 and 1:40).

After 24 h for fibroblasts and 2 h for Caco-2, cells were washed with 100 μL of HBSS (pH 7.4) and an MTT test was performed [57]. Results were expressed as percentages of viability, calculated by normalizing the absorbance measured after contact with samples with that measured after contact with CM or HBSS, representing the positive references.

##### MBE and MBE-VH Antioxidant Properties

The capability or not of both MBE and MBE-VH to protect fibroblasts and Caco-2 cells against oxidative damage was investigated.

To assess antioxidant activity on human fibroblasts, cells were pre-incubated with sample (200 µL) for 24 h and subsequently treated with H_2_O_2_ (1 mM; Carlo Erba, Milan, Italy) for 24 h [51]. As for Caco-2 cells, 2 h of pre-incubation time and 6 h of H_2_O_2_-cells contact were considered. Finally, cell substrates were washed with HBSS pH 7.4 (100 µL) and an MTT test was performed as previously described [56].

## 3. Results and Discussion

### 3.1. Choice of the Gelling Agents

**Figure 2:**
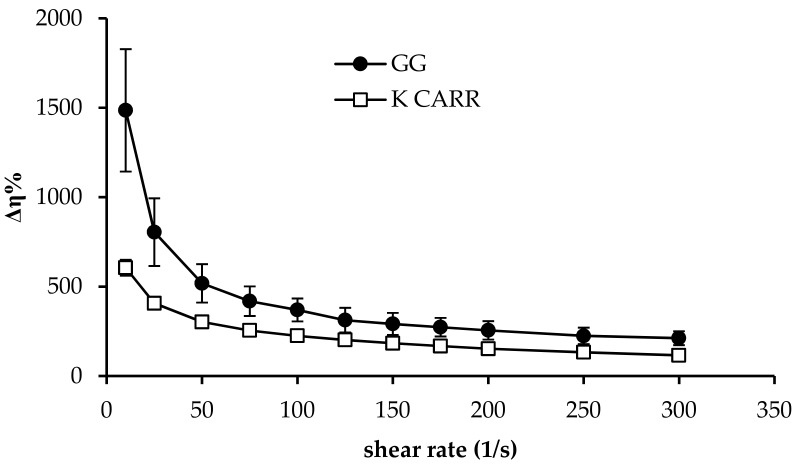
Δη% values obtained for GG an K CARR aqueous solutions (1.4% *w*/*w*) upon dilution in simulated colonic fluid (SCF) (5:2 *w*/*w*) (mean values ± SD; *n* = 3).

In Figure 2, Δη% profiles obtained for GG and K CARR aqueous solutions (1.4% *w*/*w*) upon dilution in SCF (5:2 *w*/*w*) are reported. It can be observed that, according to literature, both the two polymers are able to interact with the ions present in SCF, such as K^+^ and Na^+^. For both GG and K CARR, the complexation with monovalent and bivalent cations is responsible for the occurrence of a three-dimensional network due to coil-to-helix conformation transition, followed by helix aggregation. Hydrogen bonds with water are also involved in the gelation process [13,58]. The strength of GG-cation binding increases with increasing ionic size (Na^+^ < K^+^), the extent of aggregation, and the capability of promoting gel formation increase in the same order [58]. Additionally, for K CARR, it is reported in literature that stronger gels form in presence of K^+^ with respect to Na^+^ [59].

As shown in Figure 2, between the two polymers, GG shows a greater capacity to interact with SCF ions: dilution with SCF produces a higher increase in the viscosity of a GG solution with respect to a K CARR solution. For this reason, GG was chosen as ion-sensitive gelling agent.

**Figure 3:**
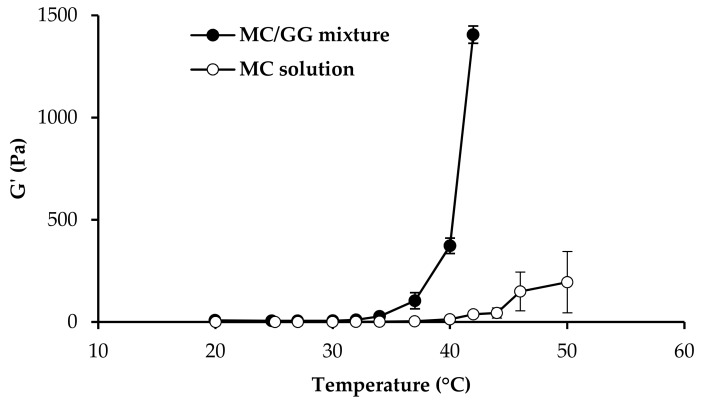
G′ values (measured at 1 Hz frequency) as a function of temperature obtained for GG 0.5% *w*/*w*/MC 0.5% *w*/*w* mixture, and MC 0.5% *w*/*w* aqueous solution (mean values ± SD; *n* = 3).

In Figure 3, G′ versus the temperature profiles of 0.5% *w*/*w* MC/0.5% *w*/*w* GG mixture, and 0.5% MC solution upon dilution in SCF, are compared. As suggested in literature, *T*_g_ is indicated by a step increase in G′ [15,60,61].

It is recognized that MC *T*_g_ is close to 50 °C and that the addition of various water-soluble polymers could affect such temperature [23].

In the present work, it seemed interesting to investigate if the addition of GG to MC could produce a decrease of MC *T*_g_. To that end, a mixture 1:1 *w*/*w* of MC and GG was subjected to oscillation measurements in comparison with MC solution prepared at the same concentration present in the mixture. Viscoelastic (oscillatory) measurements were chosen instead of viscosity ones since they provide a more accurate characterization of sample structure by subjecting it to very low and non-destructive shear stresses [62]. The presence of GG produces a lowering of MC *T*_g_, that falls within the physiological range. In fact, while for pure MC solution, a marked increase in G’ is observed for temperatures higher than 47 °C, MC/GG mixture shows a step-wise increase in G’ in the temperature range 36–40 °C. The results obtained strengthen the choice of GG as the ion-sensitive gelling agent.

### 3.2. Experimental Design

The best fit model for all the response variables (viscosity of the undiluted vehicle at 25 °C (η _25 °C_), normalized interaction parameter ΔIp, loss tangent of the vehicle diluted in SCF at 37 °C (tgδ _37 °C_), thixotropic area of the vehicle diluted in SCF at 37 °C (TA _37 °C_), and normalized work of adhesion of the vehicle diluted in SCF at 37 °C (ΔAUC/AUC _37 °C_)) of the experimental design vehicles was found to be the special cubic, except for TA _37 °C_. For said response variable, the best model was the quadratic one.

In Table 2 the mean experimental values of all the response variables considered for the seven vehicles of the experimental design are reported.

It can be observed that the vehicle 1, based on GG (0.8% *w*/*w*), is characterized by the highest value of viscosity at 25 °C, while vehicles 4–7, prepared with GG concentrations ≤ 0.4% *w*/*w*, show the lowest viscosity values. Since such a response variable is an index of sample consistency before administration, low viscosity values are preferable in order to permit an easier administration.

The interaction parameter ΔIp was used to point out the contribution of both GG (sensitive to ions) and MC (sensitive to temperature) to the increase in viscosity upon administration. As explained in the experimental part, ΔIp parameter has been calculated by subtracting the viscosity at 25 °C of the vehicle diluted in water from the viscosity at 37 °C of the same vehicle diluted in SCF. The value obtained has been normalized for the viscosity (at 25 °C) of the vehicle diluted in water. In the calculation of this parameter, viscosity at 25 °C was employed instead of that at 37 °C to highlight the eventual sensitivity of the vehicle to temperature (due to the presence of MC in mixture with GG), whereas the vehicle dilution in SCF had the aim of pointing out GG contribution. Vehicle 4, based on a mixture of GG (0.4% *w*/*w*) and MC (0.5%), is characterized by the highest value of the normalized interaction parameter ΔIp. A high value was also observed for vehicle 1 containing only GG at 0.8% *w*/*w* concentration. At 0.8 *w*/*w* concentration, the interaction of GG with SCF is very strong and able to increase vehicle viscosity seven-fold with respect to that measured at 25 °C for the vehicle diluted in water.

The viscoelastic parameter tgδ _37 °C_ was considered as an index of the structure degree of the gel formed upon administration. Vehicles 1, 2, 4, and 5 are characterized by loss tangent values lower than 1. Since such a parameter is calculated by the ratio between the loss (G″) and the storage (G′) moduli, values lower than 1 indicate a prevalence of the elastic behavior on the viscous one [63]. On the contrary, vehicles 3, 6, and 7 are characterized by a prevailing viscous behavior, showing loss tangent values higher than 1. After administration, it is desirable that a vehicle possesses higher elastic properties than viscous ones. This behavior indicates a high gel structure degree, functional to the protective action of the vehicle towards mucosa: marked elastic properties enable the gel to withstand shear stresses, undergoing elastic deformation, and thus, protecting the lesion area [53]. The results obtained indicate that HPC at 1% *w*/*w* alone (vehicle 3) does not possess a marked elastic behavior; on the other hand, MC 1% *w*/*w* (vehicle 2) and GG 0.8% *w*/*w* (vehicle 1) are characterized by optimal viscoelastic properties. It seems necessary to combine GG with HPC at concentrations higher than 0.27% *w*/*w* to obtain the desirable properties.

Additionally, TA _37 °C_ is a parameter correlated to sample structure. Positive values of TA indicate a time effect on flow. At any given shear rate or shear stress, sample viscosity continues to decrease with increasing time of shear. When shear is stopped, viscosity recovers to its initial value [62]. This behavior can be explained by the break of sample structure upon applying shear stress and by its building up when reducing the shear stress to zero. The presence of a TA should assure a reduction in sample consistency, useful for facilitating administration, and a sample structure building up with a consequent increase in consistency upon administration, functional to a protective action towards mucosa. All the vehicles containing GG, with the exception of vehicle 4, are characterized by positive values of TA _37 °C_. The highest value of such parameter was observed for vehicle 1 containing GG at 0.8% *w*/*w*.

The response variable ΔAUC/AUC _37 °C_ is an index of vehicle muchoadhesive properties. As expected, vehicle 3, containing the highest concentration of the mucoadhesive polymer HPC, is characterized by the highest mucoadhesive potential. Additionally, GG at 0.8% *w*/*w* (vehicle 1) shows good mucoadhesive properties. The lowest values of ΔAUC/AUC were observed for vehicle 2 and 4, lacking in HPC.

**Figure 4:**
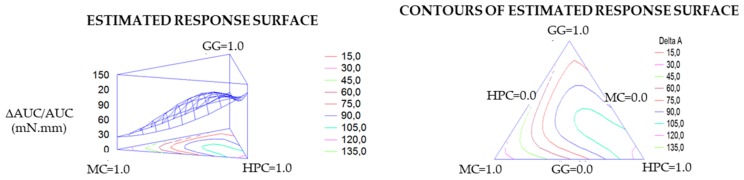
Contour plots (in tridimensional and bidimensional projections) drawn according to the best fit model for the response-variable normalized work of adhesion at 37 °C of the vehicle diluted in SCF (ΔAUC/AUC _37 °C_).

For each response variable, contour plots (in tridimensional and bidimensional projection) were drawn according to the best fit model. As an example in Figure 4, contour plots of ΔAUC/AUC are reported. The lines in each plot represent the vehicle compositions for which a same response value is predicted by the model. The individual contour plots were subsequently superimposed to identify the region of the factor space fulfilling the following constraints: η _25 °C_ ≤ 0.25 Pa·s; ΔIp ≥ 0.6 (corresponding to an increase of 60% with respect to the viscosity at room temperature of the vehicle diluted in water); tgδ _37 °C_ ≤ 1.2; TA _37 °C_ ≥ 20 Pa/s; and ΔAUC/AUC _37 °C_ ≥ 50 mN·mm. These constraints were chosen keeping in mind that the vehicle should possess, as already discussed, rheological properties suitable, at room temperature, to easy administration, and at 37 °C, for the formation of a mucoadhesive gel layer.

**Figure 5:**
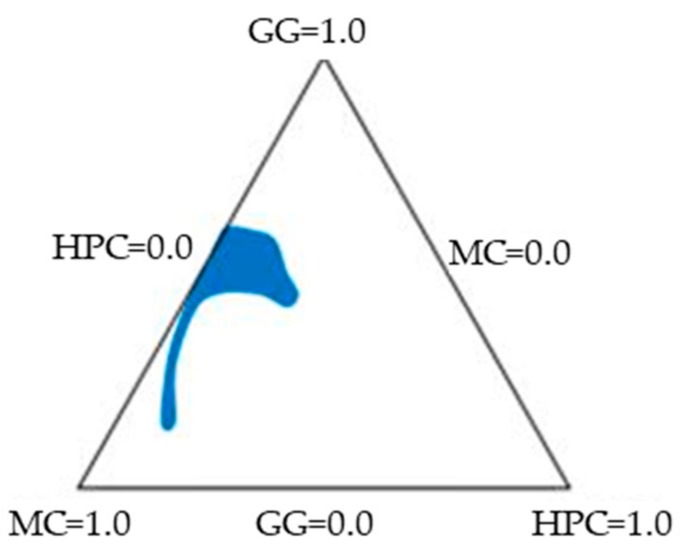
Combined contour plot showing the region of optimal mixture composition that satisfies all the constraints of the response variables.

Figure 5 shows the region of optimal composition (colorful area) that satisfies all the constraints.

The composition of the optimized vehicle (VH) (GG 0.38% *p*/*p*, MC 0.31% *p*/*p* e HPC 0.22% *p*/*p*) was chosen in this area. The results obtained from the characterization of VH were the following (mean values ± SEs; *n* = 3–5): η _25 °C_ = 0.109 ± 0.003 Pa·s; ΔIp = 0.7 ± 0.2; tgδ _37 °C_ = 0.11 ± 0.01; TA _37 °C_ = 98 ± 33 Pa/s; and ΔAUC/AUC _37 °C_ = 50 ± 10 mN·mm. In all cases, the experimental results fell inside the confidence interval of the relevant values predicted by the model, to indicate its predictive power.

### 3.3. Characterization of MBE-Loaded Optimized Vehicle (MBE-VH)

MBE-VH was characterized by the following rheological and mucoadhesive properties (mean values ± SE; *n* = 3–5): η _25 °C_ = 0.22 ± 0.02 Pa·s; ΔIp = 0.92 ± 0.06; tgδ _37 °C_ = 0.13 ± 0.02; TA _37 °C_ = 297 ± 62 Pa/s; and ΔAUC/AUC _37 °C_ = 70 ± 6 mN·mm.

It can be observed that, even though the addition of MBE into the optimized vehicle produces a significant (*p* < 0.05) increase in η _25 °C_ with respect to the vehicle as such, MBE-VH gelation properties are improved, as demonstrated, by a significant increase in ΔIp. Additionally, TA _37 °C_ and ΔAUC/AUC _37 °C_ parameters increase upon MBE addition. As already explained, this should result in an easier spreading and a longer-lasting permanence of the formulation on the mucosa. Moreover, it must be underlined that the increase in viscosity observed at 25 °C still satisfies the constraints fixed for the optimization procedure.

#### 3.3.1. Anthocyanins Assay in MBE and MBE-Loaded Optimized Vehicle (MBE-VH)

Anthocyanins in MBE were expressed as μg of C3G/mg of dried extract, considering all the areas under the curves of peaks at 520 nm. Before quantification, the RP–HPLC–PDA method was validated according to ICH guidelines. The method was linear within the range from 10 to 500 μg/mL and the correlation coefficient was higher than 0.99. To evaluate the accuracy and precision of the method, different concentrations of C3G standard solutions were analyzed in triplicate. The results indicate that the method was accurate, providing recoveries ranging from 97% to 118%, and precise, since the intraday and interday variations were lower than 1% for all the concentration levels. As far as sensibility is concerned, LOQ and LOD values determined for C3G were 10 and 3.3 μg/mL, respectively.

MBE resultantly contained 13.51 ± 0.02 (SD) μg of C3G/mg of dried weight: this content is within the range already reported in the literature for MBE [64,65]. The MBE-VH contained 61.8 ± 1.0 (SD) μg of C3G/mg of formulation. The low variability of the mean value can be considered an index of the sample homogeneity.

#### 3.3.2. In Vitro Evaluation of Biocompatibility and Antioxidant Properties of MBE-VH

##### Assessment of Fibroblast and Caco-2 Cells Viability Properties

**Figure 6:**
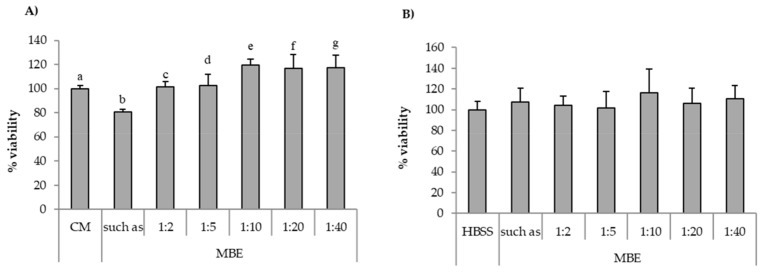
Percentage of viability values of MBE upon contact with: (**A**) fibroblasts; (**B**) Caco-2 cells. MBE 0.5% *w*/*w* solution was diluted according to different ratios (1:2, 1:5, 1:10, 1:20, and 1:40 *v*/*v*) with CM (**A**) or HBSS (**B**). CM and HBSS were used as references (mean values ± SE; *n* = 8). *t*-test (*p* < 0.05): (**A**) a versus b/e; b versus c/e/f/g.

In Figure 6A, percentage of fibroblast viability values observed for the MBE solution (0.5% *w*/*w*) diluted according to different ratios (1:40, 1:20, 1:10, 1:5, and 1:2 *v*/*v*) with complete medium (CM) are reported. It can be observed that all the samples showed percentages of viability comparable to that obtained for CM (reference), with the exception of undiluted MBE (0.5% *w*/*w*) solution, which was characterized by percentages of viability value significantly (*p* < 0.05) lower than CM. Figure 6B reports the viability results for MBE solution (0.5% *w*/*w*), diluted according to different ratios (1:40, 1:20, 1:10, 1:5, and 1:2 *v*/*v*) with HBSS, upon contact with Caco-2 cells. No significant (*p* < 0.05) differences between MBE solution/dilutions and the reference (HBSS) could be observed.

On the basis of the results obtained, MBE 1:5 and 1:2 *v/v* dilutions were chosen for the assessment of MBE antioxidant properties with both the two cell lines, as discussed in the following paragraph.

**Figure 7:**
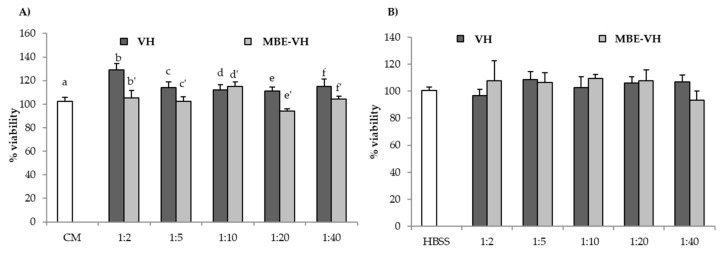
Percentage of viability values of unloaded (VH) and loaded (MBE-VH) vehicles of optimized composition observed after contact with (**A**) fibroblasts; (**B**) Caco-2 cells. Different dilutions (1:2, 1:5, 1:10, 1:20, and 1:40 *v*/*v*) in CM (A) or HBSS (B) were considered. CM and HBSS were used as references (mean values ± SE; *n* = 8). *t*-test (*p* < 0.05): (**A**) e versus e′.

Figure 7 shows percentage of cell viability values after contact (24 h for fibroblasts, 2 h for Caco-2 cells) with VH and MB-VH. Different dilutions in CM or HBSS were considered: 1:2, 1:5, 1:10, 1:20, 1:40 *v*/*v*.

VH does not present any cytotoxic effect towards both the two cell lines. In particular, in the case of fibroblasts, 1:2 *v/v* dilution is able to improve fibroblast proliferation in comparison to the reference (CM). As for MBE-VH, all the samples possess percentage of viability values similar to that of the reference CM or HBSS. Such results indicate that VH and MBE-VH at the concentration/dilution investigated do not disturb cell vitality. The gelation of unloaded and loaded formulations, induced by both temperature and ions effects, does not impair nutrient exchange between cells and the medium.

##### Assessment of MBE Antioxidant Activity

**Figure 8:**
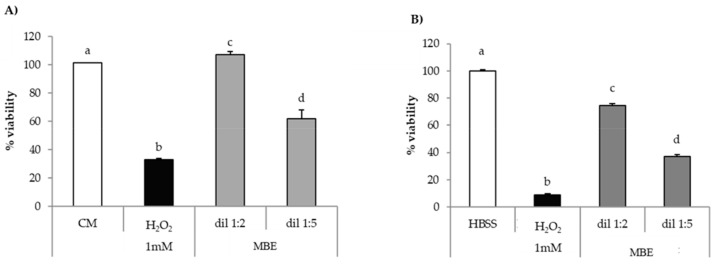
Antioxidant activity of MBE on (**A**) fibroblasts; (**B**) Caco-2 cells. CM/HBSS and H_2_O_2_ were used as references (mean values ± SE; *n* = 8). Anova one way–MRT (*p* < 0.05): (**A**) a versus b/d; b versus c/d; c versus d; (**B**) a versus b/c/d; b versus c/d; c versus d.

Figure 8A,B reports the results of in vitro evaluation of MBE antioxidant properties on fibroblasts and Caco-2 cells for MBE.

Both cell lines upon contact with H_2_O_2_ show percentage of viability values significantly (*p* < 0.05) lower than the relevant reference (CM or HBSS); these results prove the suitability of the experimental conditions (H_2_O_2_ concentration and contact time) for producing oxidative damage.

MBE is able to protect fibroblasts against oxidative stress, as indicated by percentage of viability values being higher in presence of MBE than in absence of the extract (Figure 8A). That effect was higher for the 1:2 *v/v* dilution, which was characterized by percentage of viability values comparable to that observed for untreated cells (CM). A greater dilution (1:5 *v*/*v*) corresponds to a decrease of the effect.

Additionally, for Caco-2 cells, a significant (*p* < 0.05) decrease of cell viability was observed in comparison with untreated cells (HBSS) (Figure 8B). MBE exerts a protective effect in a concentration-dependent manner against oxidative H_2_O_2_-induced damage: cell viability values for 1:2 and 1:5 dilutions were about 75% and 35%, respectively. The comparison of the results obtained for the two cell lines points out that Caco-2 cells are more sensitive to oxidative damage than fibroblasts.

##### Assessment of MBE-VH Antioxidant Properties

**Figure 9:**
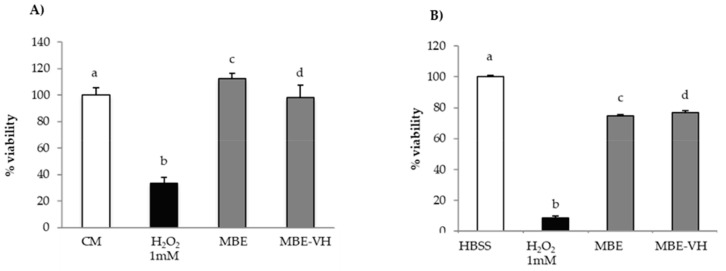
Antioxidant activity of MBE and MBE-VH on (**A**) fibroblasts; (**B**) Caco-2 cells. CM/HBSS and H_2_O_2_ were used as references (mean values ± SE; *n* = 8). Anova one way–MRT (*p* < 0.05): (**A**) a versus b; b versus c/d.; (**B**) a versus b/c/d; b versus c/d.

Figure 9A,B compares the results of in vitro evaluation of MBE and MBE-VH antioxidant properties on fibroblasts (a) and Caco-2 cells (b). MBE-VH achieves a protective effect against oxidative damage for both the cell lines considered. No statistical differences were observed between percentage of viability values obtained for the extract and after loading into vehicle. This result indicates that the vehicle does not disturb MBE activity.

## 4. Conclusions

The DoE approach permitted us to optimize the quantitative composition of a vehicle intended for colonic application via the rectal route. The vehicle was able to jellify in response to both the increase in temperature at 37 °C and the presence of SCF ions. MBE loading (0.5% *w*/*w*) into the vehicle enhanced rheological and mucoadhesive properties, which are functional for better spreading and a longer persistence of the formulation on the mucosa. Loading capacity of anthocyanins, responsible for MBE antioxidant properties, was about 90% *w*/*w* with respect to the theoretical value. Both MBE and the optimized vehicle were not cytotoxic towards both fibroblasts and Caco-2 cells. Moreover, the optimized vehicle did not disturb MBE antioxidant properties.

The overall results indicate that the optimized vehicle is a promising candidate for the local treatment of inflammatory bowel disease.

## Figures and Tables

**Table 1:** Mixture composition corresponding to the characteristic points of the Scheffè triangle.

Points of Scheffè Triangle	GG % *w*/*w*	MC % *w*/*w*	HPC % *w*/*w*
1	0.8	0	0
2	0	1	0
3	0	0	1
4	0.4	0.5	0
5	0.4	0	0.5
6	0	0.5	0.5
7	0.27	0.33	0.33

**Table 2:** Response variables of the vehicles of the simplex centroid mixture design (mean values ± SE; *n* = 3–5).

Vehicles	Response Variables				
	η _25 °C_ (Pa·s)	ΔIp	tgδ _37 °C_	TA _37 °C_ (Pa·s^−1^)	ΔAUC/AUC _37 °C_ (mN·mm)
1	0.639 ± 0.005 ^a^	6.96 ± 0.13 ^a’^	0.23 ± 0.16 ^a”^	290 ± 8 ^a^*	73 ± 22 ^a^**
2	0.258 ± 0.005 ^b^	−0.47 ± 0.05 ^b’^	0.28 ± 0.11 ^b”^	−163 ± 29 ^b^*	18 ± 4 ^b^**
3	0.378 ± 0.012 ^c^	−0.28 ± 0.06 ^c’^	3.92 ± 0.35 ^c”^	−14 ± 6 ^c^*	126 ± 18 ^c^**
4	0.1914 ± 0.0006 ^d^	7.15 ± 0.40 ^d ‘^	0.09 ± 0.02 ^d”^	−82 ± 12 ^d^*	32 ± 6 ^d^**
5	0.1842 ± 0.0006 ^e^	1.08 ± 0.14 ^e’^	0.215 ± 0.005 ^e”^	65 ± 5^e^*	79 ± 9 ^e^**
6	0.238 ± 0.001 ^f^	−0.78± 0.03 ^f’^	3.53 ± 0.14 ^f”^	23 ± 7 ^f^*	59 ± 19 ^f^**
7	0.179 ± 0.027 ^g^	−0.940 ± 0.001 ^g’^	1.42 ± 0.45 ^g”^	26 ± 3 ^g^*	95 ± 32 ^g^**

Anova one way, multiple range test, *p* < 0.05: a versus b/c/d/e/f/g; b versus c/d/e/g; c versus d/e/f/g; d versus f; e versus f; f versus g; a′ versus b′/c′/e′/f′/g′; b′ versus d′/e′; c′ versus d′/e′/g′; d′ versus e′/f/′g′; e′ versus f′/g′; a″ versus b″/c″/d″/e″/f″/g″; b″ versus f″/g″; c″ versus f″/g″; a* versus b*/c*/d*/e*/f*/g*; b* versus c*/d*/e*/f*/g*; c* versus d*/e*; d* versus e*/f*/g*; e* versus f*; b** versus c**/e**/g**; c** versus d**/f**; d** versus g**.

## References

[1] Lautenschläger C., Schmidt C., Fischer D., Stallmach A. (2014). Drug delivery strategies in the therapy of inflammatory bowel disease. Adv. Drug Deliv. Rev..

[2] Hanauer S.B. (2006). Inflammatory bowel disease: Epidemiology, pathogenesis, and therapeutic opportunities. Inflamm. Bowel Dis..

[3] Lichtenstein G.R., Rutgeerts P. (2010). Importance of mucosal healing in ulcerative colitis. Inflamm. Bowel Dis..

[4] Garber A., Regueiro M. (2019). Extraintestinal Manifestations of Inflammatory Bowel Disease: Epidemiology, Etiopathogenesis, and Management. Curr. Gastroenterol. Rep..

[5] Taylor K.M., Irving P.M. (2011). Optimization of conventional therapy in patients with IBD. Nat. Rev. Gastroenterol. Hepatol..

[6] Shahdadi Sardo H., Saremnejad F., Bagheri S., Akhgari A., Afrasiabi Garekani H., Sadeghi F. (2019). A review on 5-aminosalicylic acid colon-targeted oral drug delivery systems. Int. J. Pharm..

[7] Neurath M.F., Travis S.P.L. (2012). Mucosal healing in inflammatory bowel diseases: A systematic review. Gut.

[8] Sharma S., Sinha VR. (2018). Current pharmaceutical strategies for efficient site specific delivery in inflamed distal intestinal mucosa. J. Control. Release.

[9] Pásztor E., Makó Á., Csóka G., Fenyvesi Z., Benko R., Prosszer M., Marton S., Antal I., Klebovich I. (2011). New formulation of in situ gelling Metolose-based liquid suppository. Drug Dev. Ind. Pharm..

[10] Yuan Y., Ying C., Li Z., Hui-ping Z., Yi-Sha G., Bo Z., Xia H., Ling Z., Xiao-hui W., Li C. (2012). Thermosensitive and mucoadhesive in situ gel based on poloxamer as new carrier for rectal administration of nimesulide. Int. J. Pharm..

[11] Deng H., Dong A., Song J., Chen X. (2019). Injectable thermosensitive hydrogel systems based on functional PEG/PCL block polymer for local drug delivery. J. Control. Release.

[12] Wang Q., Zuo Z., Cheung C.K.C., Leung S.S.Y. (2019). Updates on thermosensitive hydrogel for nasal, ocular and cutaneous delivery. Int. J. Pharm..

[13] Vigani B., Faccendini A., Rossi S., Sandri G., Bonferoni M.C., Gentile M., Ferrari F. (2019). Development of a mucoadhesive and in situ gelling formulation based on κ-carrageenan for the treatment of the oral mucositis. I. A functional in vitro characterization. Mar. Drugs.

[14] Vigani B., Rossi S., Gentile M., Sandri G., Bonferoni M.C., Cavalloro V., Martino E., Collina S., Ferrari F. (2019). Development of a Mucoadhesive and an in Situ Gelling Formulation Based on k-Carrageenan for Application on Oral Mucosa and Esophagus Walls. II. Loading of a Bioactive Hydroalcoholic Extract. Mar. Drugs.

[15] Rossi S., Ferrari F., Bonferoni M.C., Sandri G., Faccendini A., Puccio A., Caramella C. (2014). Comparison of poloxamer- and chitosan-based thermally sensitive gels for the treatment of vaginal mucositis. Drug Dev. Ind. Pharm..

[16] Ward M.A., Georgiou T.K. (2011). Thermoresponsive polymers for biomedical applications. Polymers.

[17] Ruel-Gariépy E., Leroux J.C. (2004). In situ-forming hydrogels—Review of temperature-sensitive systems. Eur. J. Pharm. Biopharm..

[18] Schmaljohann D. (2006). Thermo- and pH-responsive polymers in drug delivery. Adv. Drug Deliv. Rev..

[19] Singh B., Khurana R.K., Garg B., Saini S., Kaur R. (2017). Stimuli-Responsive Systems with Diverse Drug Delivery and Biomedical Applications: Recent Updates and Mechanistic Pathways. Crit. Rev. Ther. Drug Carr. Syst..

[20] Klouda L. (2015). Thermoresponsive hydrogels in biomedical applications: A seven-year update. Eur. J. Pharm. Biopharm..

[21] Lodge T.P., Maxwell A.L., Lott J.R., Schmidt P.W., McAllister J.W., Morozova S., Bates F.S., Li Y., Sammler R.L. (2018). Gelation, Phase Separation, and Fibril Formation in Aqueous Hydroxypropylmethylcellulose Solutions. Biomacromolecules.

[22] Vigani B., Faccendini A., Rossi S., Sandri G., Bonferoni M.C., Grisoli P., Ferrari F. (2019). Development of a mucoadhesive in situ gelling formulation for the delivery of *Lactobacillus gasseri* into vaginal cavity. Pharmaceutics.

[23] Shimokawa K., Saegusa K., Ishii F. (2009). Rheological properties of reversible thermo-setting in situ gelling solutions with the methylcellulose–polyethylene glycol–citric acid ternary system (2): Effects of various water-soluble polymers and salts on the gelling temperature. Colloids Surf. B Biointerfaces.

[24] Makó Á., Csóka G., Pásztor E., Marton S., Horvai G., Klebovich I. (2009). Formulation of thermoresponsive and bioadhesive gel for treatment of oesophageal pain and inflammation. Eur. J. Pharm. Biopharm..

[25] Forghani A., Devireddy R. (2018). Methylcellulose Based Thermally Reversible Hydrogels. Methods Mol. Biol..

[26] Demir Oğuz Ö., Ege D. (2018). Rheological and Mechanical Properties of Thermoresponsive Methylcellulose/Calcium Phosphate-Based Injectable Bone Substitutes. Materials.

[27] Van Tomme S.R., Storm G., Hennink W.E. (2008). In situ gelling hydrogels for pharmaceutical and biomedical applications. Int. J. Pharm..

[28] Parekh H.B., Rishad J., Jivani N.P., Patel L.D., Makwana A., Sameja K. (2012). Novel in situ polymeric drug delivery system: A review. J. Drug Deliv. Ther..

[29] Adrover A., Paolicelli P., Petralito S., Di Muzio L., Trilli J., Cesa S., Tho I., Casadei M.A. (2019). Gellan Gum/Laponite Beads for the Modified Release of Drugs: Experimental and Modeling Study of Gastrointestinal Release. Pharmaceutics.

[30] Yoshioka Y., Akiyama H., Nakano M., Shoji T., Kanda T., Ohtake Y., Takita T., Matsuda R., Maitani T. (2008). Orally administered apple procyanidins protect against experimental inflammatory bowel disease in mice. Int. Immunopharmacol..

[31] Piberger H., Oehme A., Hofmann C., Dreiseitel A., Sand P.G., Obermeier F., Schoelmerich J., Schreier P., Krammer G., Rogler G. (2011). Bilberries and their anthocyanins ameliorate experimental colitis. Mol. Nutr. Food Res..

[32] Biedermann L., Mwinyia J., Scharla M., Freia P., Zeitzc J., Kullak-Ublickb G.A., Vavrickaa S.R., Frieda M., Webere A., Humpff H.U. (2013). Bilberry ingestion improves disease activity in mild to moderate ulcerative colitis—An open pilot study. J. Crohn’s Colitis.

[33] Farzaei H.M., Rahimi R., Abdollahi M. (2015). The role of dietary polyphenols in the management of inflammatory bowel disease. Curr. Pharm. Biotechnol..

[34] Barbalho S.M., Bosso H., Salzedas-Pescinini L.M., de Alvares Goulart R. (2018). Green tea: A possibility in the therapeutic approach of inflammatory bowel diseases? Green tea and inflammatory bowel diseases. J. Tradit. Complement. Med..

[35] Nunes C., Freitas V., Almeida L., Laranjinha J. (2019). Red wine extract preserves tight junctions in intestinal epithelial cells under inflammatory conditions: Implications for intestinal inflammation. Food Funct..

[36] Suwalsky M., Vargas P., Avello M., Villena F., Sotomayor C.P. (2008). Human erythrocytes are affected in vitro by flavonoids of *Aristotelia chilensis* (Maqui) leaves. Int. J. Pharm..

[37] Rubilar M., Jara C., Poo Y., Acevedo F., Gutierrez C., Sineiro J., Shene C. (2011). Extracts of Maqui (*Aristotelia chilensis*) and Murta (*Ugnimolinae Turcz.*): Souces of antioxidant compounds and α-glucosidase/α-amylase inhibitors. J. Agric. Food Chem..

[38] Benatrehina P.A., Pan L., Naman C.B., Li J., Kinghorn A.D. (2018). Usage, biological activity, and safety of selected botanical dietary supplements consumed in the United States. J. Tradit. Complement. Med..

[39] Di Lorenzo A., Sobolev A.P., Nabavi S.F., Sureda A., Moghaddam A.H., Khanjani S., Di Giovanni C., Xiao J., Shirooie S., Sokeng A.J.T. (2019). Antidepressive effects of a chemically characterized maqui berry extract (*Aristotelia chilensis* (molina) stuntz) in a mouse model of Post-stroke depression. Food Chem. Toxicol..

[40] Céspedes C.L., El-Hafidi M., Pavon N., Alarcon J. (2008). Antioxidant and cardioprotective activities of phenolic extracts from fruits of Chilean blackberry *Aristotelia chilensis* (Elaeocarpaceae), Maqui. Food Chem..

[41] Schreckinger M., Wang J., Yousef G., Lila M., De Mejia E. (2010). Antioxidant capacity and in vitro inhibition of adipogenesis and inflammation by phenolic extracts of Vacciniumfloribundum and *Aristotelia chilensis*. J. Agric. Food Chem..

[42] Mølgaard P., Holler J.G., Asar B., Liberna I., Rosenbæk L.B., Jebjerg C.P., Jørgensen L., Lauritzen J., Guzman A., Adsersen A. (2011). Antimicrobial evaluation of Huilliche plant medicine used to treat wounds. J. Ethnopharmacol..

[43] Genskowsky E., Puente L.A., Pérez-Álvarez J.A., Fernández-López J., Muñoz L.A., Viuda-Martos M. (2016). Determination of polyphenolic profile, antioxidant activity and antibacterial properties of maqui [*Aristotelia chilensis* (Molina) Stuntz] a Chilean blackberry. J. Sci. Food Agric..

[44] Campieri M., Corbelli C., Gionchetti P., Brignola C., Belluzzi A., Di Febo G., Zagni P., Brunetti G., Miglioli M., Barbara L. (1992). Spread and Distribution of 5-ASA Colonic Foam and 5-ASA Enema in Patients with Ulcerative Colitis. Dig. Dis. Sci..

[45] Zahir-Jouzdani F., Wolf J.D., Atyabi F., Bernkop-Schnürch A. (2018). In situ gelling and mucoadhesive polymers: Why do they need each other?. Expert Opin. Drug Deliv..

[46] Schiller C., Frohlich C.-P., Giessmann T., Siegmund W., Monnikes H., Hosten N., Weitschies W. (2005). Intestinal fluid volumes and transit of dosage forms as assessed by magnetic resonance imaging. Aliment. Pharmacol. Ther..

[47] Marques M.R.C., Loebenberg R., Almukainzi M. (2011). Simulated biological fluids with possible application in dissolution testing. Dissolut. Technol..

[48] Dejagher B., Vander Heyden Y. (2011). Experimental designs and their recent advances in set-up, data interpretation, and analytical applications. J. Pharm. Biomed. Anal..

[49] Tenci M., Rossi S., Bonferoni M.C., Sandri G., Mentori I., Boselli C., Cornaglia A.I., Daglia M., Marchese A., Caramella C. (2017). Application of DoE approach in the development of mini-capsules, based on biopolymers and manuka honey polar fraction, as powder formulation for the treatment of skin ulcers. Int. J. Pharm..

[50] Vigani B., Rossi S., Sandri G., Bonferoni M.C., Milanesi G., Bruni G., Ferrari F. (2018). Coated electrospun alginate-containing fibers as novel delivery systems for regenerative purposes. Int. J. Nanomed..

[51] Hibbert D.B. (2012). Experimental design in chromatography: A tutorial review. J. Chromatogr. B.

[52] Furlanetto S., Cirri M., Piepel G., Mennini N., Mura P. (2011). Mixture experiment methods in the development and optimization of microemulsion formulations. J. Pharm. Biomed. Anal..

[53] Mori M., Rossi S., Ferrari F., Bonferoni M.C., Sandri G., Chlapanidas T., Torre M.L., Caramella C. (2016). Sponge-like dressings based on the association of chitosan and sericin for the treatment of chronic skin ulcers. I. Design of experiments-assisted development. J. Pharm. Sci..

[54] Sandri G., Rossi S., Ferrari F., Bonferoni M.C., Zerrouk N., Caramella C. (2004). Mucoadhesive and penetration enhancement properties of three grades of hyaluronic acid using porcine buccal and vaginal tissue, Caco-2 cell lines, and rat jejunum. J. Pharm. Pharmacol..

[55] Draper N.R., Smith H. (1981). Applied Regression Analysis.

[56] Cornell J.A., Bloomfield P., Cressie N.A.C., Fisher N.I., Johnstone I.M., Kodane J.R., Ryan L.M., Scott D.W., Silverman B.W., Smith A.F.M., Tengels J.L. (2002). The original mixture problem: Design and models for exploring the entire simplex factor space. Experiments with Mixtures: Designs, Models, and the Analysis of Mixture Data.

[57] Tenci M., Rossi S., Bonferoni M.C., Sandri G., Boselli C., Di Lorenzo A., Daglia M., Icaro Cornaglia A., Gioglio L., Perotti C. (2016). Pectin/chitosan particles for the delivery of platelet lysate and manuka honey in chronic skin ulcers. Int. J. Pharm..

[58] Morris E.R., Nishinari K., Rinaudo M. (2012). Gelation of gellan—A review. Food Hydrocoll..

[59] Daniel-da-Silva A.L., Ferreira L., Gil A.M., Trindade T. (2011). Synthesis and swelling behavior of temperature responsive κ-carrageenan nanogels. J. Colloid Interface Sci..

[60] Aka-Any-Grah A., Bouchemal K., Koffi A., Zhang M., Djabourov M., Ponchel G. (2010). Formulation of mucoadhesive vaginal hidrogels insensitive to dilution with vaginal fluids. Eur. J. Pharm. Biopharm..

[61] Caramella C.M., Rossi S., Ferrari F., Bonferoni M.C., Sandri G. (2015). Mucoadhesive and thermogelling systems for vaginal drug delivery. Adv. Drug Deliv. Rev..

[62] Tadros T. (2004). Application of rheology for assessment and prediction of the long-term physical stability of emulsions. Adv. Colloid Interface Sci..

[63] Mori M., Rossi S., Ferrari F., Bonferoni M.C., Sandri G., Riva F., Tenci M., Del Fante C., Nicoletti G., Caramella C. (2016). Sponge-Like Dressings Based on the Association of Chitosan and Sericin for the Treatment of Chronic Skin Ulcers. II. Loading of the Hemoderivative Platelet Lysate. J. Pharm. Sci..

[64] Fredes C., Yousef G.G., Robert P., Grace M.H., Lila M.A., Gómez M., Montenegro G. (2014). Anthocyanin profiling of wild maqui berries (*Aristotelia chilensis* [Mol.] Stuntz) from different geographical regions in Chile. J. Sci. Food Agric..

[65] Brauch J.E., Buchweitz M., Schweiggert R.M., Carle R. (2016). Detailed analyses of fresh and dried maqui (*Aristotelia chilensis* (Mol.) Stuntz) berries and juice. Food Chem..

